# Understanding and engineering alcohol-tolerant bacteria using OMICS technology

**DOI:** 10.1007/s11274-018-2542-4

**Published:** 2018-10-19

**Authors:** Takaaki Horinouchi, Tomoya Maeda, Chikara Furusawa

**Affiliations:** 1 Center for Biosystems Dynamics Research (BDR), RIKEN, 6-2-3 Furuedai, Suita, Osaka 565-0874 Japan; 2 Universal Biology Institute, The University of Tokyo, 7-3-1 Hongo, Tokyo, 113-0033 Japan

**Keywords:** Adaptive laboratory evolution, Alcohol tolerance, Bacteria, Omics technology

## Abstract

Microbes are capable of producing alcohols, making them an important source of alternative energy that can replace fossil fuels. However, these alcohols can be toxic to the microbes themselves, retaring or inhibiting cell growth and decreasing the production yield. One solution is improving the alcohol tolerance of such alcohol-producing organisms. Advances in omics technologies, including transcriptomic, proteomic, metabolomic, and genomic technologies, have helped us understand the complex mechanisms underlying alcohol toxicity, and such advances could assist in devising strategies for engineering alcohol-tolerant strains. This review highlights these advances and discusses strategies for improving alcohol tolerance using omics analyses.

## Introduction

Engineering microorganisms to produce biochemicals has attracted attention as a strategy for reducing the dependency on fossil fuels and developing alternate renewable energy sources. Microbial production of alcohols such as ethanol, propanol, butanol, and other short-chain alcohols has been refined by many researchers (Hanai et al. 2007; Keasling and Chou 2008; Stephanopoulos 2008; Gronenberg et al. 2013). The selection of a microbial production host for an industrial biotechnology process is primarily determined by its potential to efficiently produce the product of interest. Yeast and bacteria are frequently selected as host organisms for bioalcohol production (Lau et al. 2010; Yamamoto et al. 2013). The yeast *Saccharomyces cerevisiae* is a natural producer of ethanol that has been widely applied for the production of bioethanol. Conversely, bacteria have advantages such as rapid growth, the utilization of various carbon sources, and the availability of genetic and molecular tools (Rumbold et al. 2009; Koppolu and Vasigala 2016).

In the bioproduction of these alcohols, a major problem is that the toxicity of the alcoholic compounds slows or inhibits cell growth, decreasing the production yield. The alcohol tolerance of bacteria is generally inferior to that of yeast; thus, alcohol toxicity is a more serious problem for bioproduction using bacteria. One strategy to overcome this problem is to develop strains that have tolerance to the target compounds. Thus, it is important to understand the mechanisms underlying alcohol tolerance.

Bacterial alcohol stress response has been studied for more than 40 years, and the physiological effect of alcohol stress has been well described. These studies primarily investigated the mechanisms by which alcoholic compounds affect the bacterial membrane. For example, alcohols interact directly with the lipid bilayer because of their amphiphilicity, and membrane fluidity is altered by the insertion of alcohols into cellular membranes (Ingram 1976). These changes in fluidity increase membrane permeability and induce conformational changes in membrane proteins, and ethanol-induced membrane changes induce the expression of heat-shock and phage-shock proteins (Neidhardt et al. 1984; Brissette et al. 1990). Alcoholic compounds also cause the partial breakdown of membrane function. This membrane damage causes various perturbations to cells such as ion leakage or loss of energy. In this context, many studies have focused on the relationship between alcohol tolerance and membrane composition. For example, changes of fatty acid composition (increase in the amount of unsaturated fatty acids) are observed during adaptation to ethanol in *Escherichia coli* (Ingram 1976; Berger et al. 1980). As another example, modifications of the unsaturated/saturated fatty acid ratio are found in *Clostridium acetobutylicum* cell membranes during acetone-butanol fermentation (Lepage et al. 1987). Modification of membrane composition via genetic manipulation also confers alcohol tolerance (Grandvalet et al. 2008; Luo et al. 2009). These studies illustrate that modifying the membrane composition can partially mitigate the toxicity of alcohols.

Alcoholic compounds activate various stress response networks (Bury-Moné et al. 2009). For example, the regulatory mechanisms of envelope stress (Ades 2004), oxidative stress (Belkin et al. 1996), and the respiratory cycle (Garbe and Yukawa 2001) are affected by alcohols. These responses are induced by membrane damage and physiological changes of the cellular state (e.g., changes of membrane fluidity, protein misfolding, ion leakage). To establish a rational strategy for improving bacterial alcohol tolerance, it is necessary to understand the cellular activities related to alcohol toxicity. We believe that advances in omics technologies, including transcriptomic, proteomic, metabolomic, and genomic technologies, can help us understand the impact of bacterial alcohol stress.

**Fig. 1:**
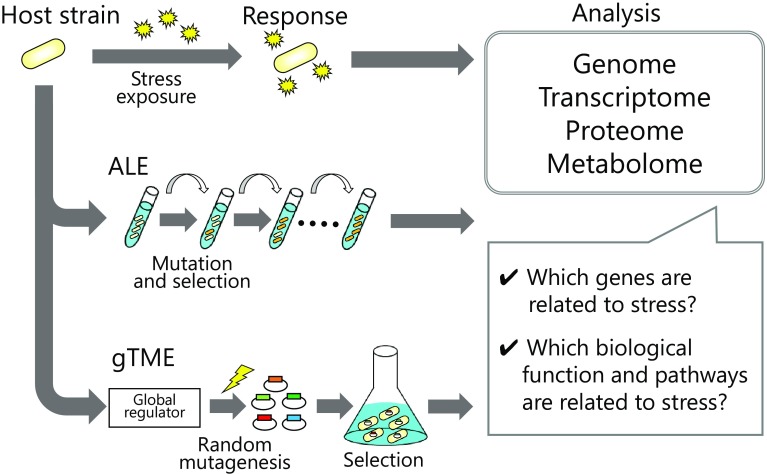
Strategy for the understanding alcohol-tolerance using omics technologies and recent engineering approaches for strain improvement. Adaptive laboratory evolution (ALE) is an approach for generating cells with improved growth and stress tolerance by mutations and natural selection. Global transcription machinery engineering (gTME) is an approach for obtaining various cellular phenotypes by reprogramming gene transcription using error-prone PCR. To combine omics analyses with these approaches, it is possible to expand our research for phenotypes of alcohol tolerance

This review highlights advances in the use of omics technologies to understand alcohol tolerance in bacteria. First, we describe the comprehensive effect of alcohol toxicity using omics technologies. Further, we focus on several approaches for improving alcohol tolerance. We also focus on the alcohol stress response and tolerance of several bacterial species. *E. coli* has been used for biofuel production by engineering production pathways (Clomburg and Gonzalez 2010; Peralta-Yahya and Keasling 2010), and its well-characterized genetic background and well-developed genetic tools allow for flexible and economical process design for large-scale alcohol production. Likewise, *C. acetobutylicum* has been used for decades to produce butanol (Jones et al. 1982; Hermann et al. 1985). Most recently, cyanobacteria have attracted attention as promising industrial microorganisms for bioproduction because the cells can directly fix atmospheric carbon dioxide and convert it to a target compound using energy from photosynthesis (Nozzi et al. 2013; Lau et al. 2015). In this study, we review studies on alcohol tolerance in these species with an emphasis on improving alcohol production. Furthermore, to combine omics approaches with recent engineering approaches for strain improvement, it is possible to expand our search for phenotypes of alcohol tolerance (Fig. 1). We describe these promising approaches toward understanding and improving microbial tolerance to alcohol.

## Understanding alcohol tolerance using omics technologies

Alcoholic compounds activate various stress response networks by causing membrane damage. Comprehensive measurements made using omics technologies help us analyze the effect of alcohol stress on cellular states. Several studies provided lists of genes, proteins, and intracellular compounds that participate the alcohol stress response. For example, to screen genes related to this stress response, investigators analyzed gene expression in the presence and absence of alcohols in the culture medium. These findings should be a starting point for understanding the molecular mechanisms involved in ethanol stress tolerance, and thus, they represent fundamental knowledge for designing ethanol-tolerant cells. Recent studies on the bacterial alcohol stress response are summarized in Table 1.

**Table 1:** Omics experiments with bacteria

Species	Strain	Stress	Analysis	References
*E. coli*	Ethanol-tolerant strain LY01	Ethanol	Transcriptome	Gonzalez et al. (2003)
*E. coli*	Transposon library, overexpression library	Ethanol	Enrichment	Goodarzi et al. (2010)
*E. coli*	Ethanol-evolved strains A–F	Ethanol	Transcriptome	Horinouchi et al. (2010)
*E. coli*	Mutated IrrE from *Deinococcus radiodurans*	Ethanol	Transcriptome, proteome	Chen et al. (2012)
*E. coli*	Fosmid library	Ethanol	Enrichment	Nicolaou et al. (2012)
*E. coli*	trans10	Ethanol, *n*-butanol, Isobutanol	Metabolome	Wang et al. (2013)
*E. coli*	Genomic library	Ethanol	Enrichment analysis, transcriptome, proteome	Woodruff et al. (2013)
*E. coli*	Tolerant strain MTA156, MTA157, and MTA160	Ethanol	DNAseq, RNAseq, ribosome profiling	Haft et al. (2014)
*E. coli*	Genomic library of solvent-tolerant *Lactobacillus plantarum*	Ethanol	Enrichment	Zingaro et al. (2014)
*E. coli*	Metagenomic and heterologous genomic libraries of sigma factor	Ethanol	Enrichment, transcriptome	Gaida et al. (2015)
*E. coli*	Ethanol-evolved strains A–F	Ethanol	Genome, transcriptome, metabolome	Horinouchi et al. (2015)
*E. coli*	High tolerance populations HT1-16	Ethanol	Genome	Swings et al. (2017)
*E. coli*	Ethanol-tolerant mutant EM	Ethanol	Genome	Chen et al. (2018)
*E. coli*	Ethanol-tolerant ethanologenic strains	Ethanol	Genome	Lupino et al. (2018)
*E. coli*	IPA-tolerant strains A–F	Isopropanol	Genome, transcriptome	Horinouchi et al. (2017a)
*E. coli*	DH1	*n*-Butanol	Transcriptome, proteome	Rutherford et al. (2010)
*E. coli*	Efflux pump library	*n*-Butanol	Enrichment	Dunlop et al. (2011)
*E. coli*	CRP mutation library	*n*-Butanol	Transcriptome	Lee et al. (2011)
*E. coli*	Genomic library, overexpression library	*n*-Butanol	Enrichment	Reyes et al. (2011)
*E. coli*	Adaptive mutants MG1–6 and MY1–4	*n*-Butanol	Genome, transcriptome	Reyes et al. (2012)
*E. coli*	Adapted strains B500, G500, O500, H500, and P500	*n*-Butanol and 3 abiotic stress	Genome, transcriptome, cross-stress tolerance	Dragosits et al. (2013)
*E. coli*	Transposon library	*n*-Butanol and 12 chemicals	Enrichment, transcriptome	Rau et al. (2016)
*E. coli*	Sigma70 mutant	*n*-Butanol	Transcriptome	Si et al. (2016)
*E. coli*	Fifty-five evolved strains	*n-*Butanol and ten chemicals	Genome, transcriptome, cross-stress tolerance	Horinouchi et al. (2017b)
*E. coli*	Butanol-tolerant evolved strain PKH5000	*n*-Butanol	Transcriptome, phenotype microarray	Jeong et al. (2017)
*E. coli*	BW25113 and knockout strains	Isobutanol	Transcriptome	Brynildsen and Liao (2009)
*E. coli*	Isobutanol-tolerant mutant SA481	Isobutanol	Genome	Atsumi et al. (2010)
*E. coli*	Isobutanol-tolerant clone G3.2, X3.5	Isobutanol	Genome, transcriptome	Minty et al. (2011)
*E. coli*	CRP mutation library	Isobutanol	Transcriptome	Chong et al. (2014)
*C. acetobutylicum*	*groESL* overexpression strain	*n*-Butanol	Transcriptome	Tomas et al. (2004)
*C. acetobutylicum*	*spo0A* disrupt mutant	*n*-Butanol	Transcriptome	Tomas et al. (2003a)
*C. acetobutylicum*	*groESL* overexpression strain	*n*-Butanol	Transcriptome, metabolic flux	Tomas et al. (2003b)
*C. acetobutylicum*	Genomic library	*n*-Butanol	Enrichment	Borden and Papoutsakis (2007)
*C. acetobutylicum*	ATCC824	*n*-Butanol, butyrate, acetate	Transcriptome	Alsaker et al. (2010)
*C. acetobutylicum*	Butanol-tolerant mutant Rh8	*n*-Butanol	Proteome	Mao et al. (2010)
*C. acetobutylicum*	DSM1731	*n*-Butanol	Proteome	Jia et al. (2012)
*C. acetobutylicum*	Butanol-tolerant mutant Rh8	*n*-Butanol	Genome, proteome	Bao et al. (2014)
*C. acetobutylicum*	ATCC824	*n*-Butanol	Metabolome	Wang et al. (2016)
*C. acetobutylicum*	Butanol-tolerant asporogenic strain JB200	*n*-Butanol	Genome	Xu et al. (2017)
*Synechocystis*	sp. PCC 6803	Ethanol	Proteome	Qiao et al. (2012)
*Synechocystis*	Δ*sll0794*	Ethanol	Proteome	Song et al. (2014)
*Synechocystis*	sp. PCC 6803	Ethanol	Metabolome	Zhu et al. (2015)
*Synechocystis*	sp. PCC 6803	Ethanol, *n*-butanol, hexane, salt	sRNA-seq	Pei et al. (2017)
*Synechocystis*	Recombinant strains UL 004 and UL 030	Ethanol	Flow cytometry	Lopes da Silva et al. (2018)
*Synechocystis*	Tolerant mutant SY1043	Isopropanol	Genome, single-cell screening	Hirokawa et al. (2018)
*Synechocystis*	sp. PCC 6803	*n*-Butanol	Transcriptome	Anfelt et al. (2013)
*Synechocystis*	Evolved strain S1, S3, S4	*n*-Butanol	Metabolome	Wang et al. (2014)
*Synechocystis*	∆*slr1037* mutant	*n*-Butanol	Metabolome	Niu et al. (2015)
*Synechocystis*	Evolved strain T(1)–T(4)	Isobutanol	Genome	Matsusako et al. (2017)

Transcriptome analyses of *E. coli* under isobutanol stress revealed that cellular functions related to respiration, phosphate metabolism, and iron metabolism were perturbed (Brynildsen and Liao 2009). Further investigation via network component and knockout analyses illustrated that several transcription factors play important roles in the isobutanol response network. This study proposed that the isobutanol stress response is triggered by a malfunction of quinone (Brynildsen and Liao 2009). As another example, transcriptome and proteome analyses of *E. coli* exposed to *n*-butanol demonstrated that this stress activates several stress response machineries simultaneously, including cell envelope stress, oxidative stress, and acid stress responses. This stress also induces protein misfolding and activates efflux systems (Rutherford et al. 2010). These analyses allowed us to identify key genes involved in alleviating oxidative stress, protein misfolding, and other causes of growth defects. The genes and biological activities identified in these studies could be important assets for engineering alcohol-tolerant bacteria.

In *E. coli*, metabolomic analyses of its responses to ethanol, *n*-butanol, and isobutanol stresses revealed that several amino acids and osmoprotectants, such as isoleucine, valine, glycine, glutamate, and trehalose, are key metabolites that protect against these stresses (Wang et al. 2013). Several transcriptomic studies also demonstrated the relationship between alcohol stress and these low-molecular-weight compounds (Gonzalez et al. 2003; Horinouchi et al. 2010, 2017a; Swings et al. 2017). Although the roles of these compounds in the alcohol stress response remain unclear, they may mitigate the growth inhibition caused by alcohol stress. Further, alcohol can inhibit translation, and this inhibition can be mitigated by the addition of exogenous amino acids or deletion of repressors (Haft et al. 2014). Specifically, Haft et al. (2014) found that ethanol has detrimental effects on translational misreading, ribosome stalling, and the aberrant termination of polypeptide synthesis. They demonstrated that such effects on translation are caused by methionine depletion.

Transcriptomic analyses of *C. acetobutylicum* under *n*-butanol stress identified genes related to the *n*-butanol stress response (Tomas et al. 2003a, b, 2004). These studies identified important roles of the *groESL* chaperone system and the global regulator of sporulation *spo0A*. Further analyses suggested that the expression level of genes related to the functional categories of “glycolysis”, “amino acid biosynthesis and transport”, and “oxidative stress response” are changed by exposure to *n*-butanol stress (Alsaker et al. 2010). Although significant physiological differences exist between *E. coli* and *C. acetobutylicum* (such as cellular membrane structure, composition, and the ability to form spores), similar functional categories were noted (Alsaker et al. 2010). Although differences of cellular membrane structure strongly affect *n*-butanol tolerance, these similarities of functions response to *n*-butanol suggest that a common mechanism of alcohol toxicity exists between these species.

The alcohol stress responses of *Synechocystis* sp. have been analyzed using omics technologies since 2010 (summarized in Table 1). Several studies revealed that oxidative stress response is activated by alcohols. For example, transcriptomic analysis of *Synechocystis* spp. exposed to *n*-butanol demonstrated that oxidative stress response-related genes were upregulated (Anfelt et al. 2013). As another example, proteomic analysis of *Synechocystis* revealed that oxidative stress response is induced by ethanol (Qiao et al. 2012). Photosynthetic organisms may be more susceptible to the effects of alcohols because of the sensitivity to the redox state of key molecules such as plastoquinone and the intricate organization of the membrane-bound photosynthetic apparatus. Alleviating oxidative stress is a target for improving the alcohol tolerance of *Synechocystis*.

## Adaptive laboratory evolution (ALE) of alcohol tolerance

Adaptive laboratory evolution is a powerful tool for analyzing phenotypic and genotypic changes during bacterial evolution (Dragosits and Mattanovich 2013; Winkler and Kao 2014). In this approach, cells are cultured under a selective environment for many generations, leading to adaptive evolution. Then, using omics technologies, we can obtain genome-wide information about adaptive phenotypic and genotypic changes resulting from the selective pressure. Advances in omics technologies, especially decreases in the cost of genome re-sequencing, have made ALE a standard approach for investigating and engineering desired phenotypes and analyzing alcohol tolerance in various microorganisms (summarized in Table 1).

In some cases, different studies identified different genes as contributing to alcohol tolerance even though the studies used the same stressor for ALE experiments. For example, Minty et al. (2011) identified mutations in *mdh* (malate dehydrogenase) and *rph* (defective ribonuclease PH) in *E. coli* evolved under isobutanol stress (Minty et al. 2011). However, neither change was found in a study of isobutanol-tolerant *E. coli* by Atsumi et al. (2010). Alternatively, mutations in *tnaA* (tryptophanase) and *yhbJ* (renamed as *rapZ*, RNase adaptor protein) identified by Atsumi et al. (2010) were not identified by Minty et al. (2011). These discordances may be caused by differences in experimental conditions, such as the parental strain, culture conditions, and selective pressure. Another possible cause is the diversity of the obtained alcohol-tolerant strains. Many different mutations are often acquired among the evolved strains obtained via multiple ALEs under the same experimental conditions. It is important to verify whether the identified mutations contribute to alcohol tolerance.

When bacterial cells become tolerant to one stress via ALE, they sometimes also become more tolerant to other stresses, a phenomenon called cross protection. However, they sometimes become more sensitive to other stresses, a phenomenon called collateral sensitivity. These scenarios provide valuable information regarding the mechanisms of stress tolerance. Notably, the existence of cross protection and collateral sensitivity among *n*-butanol and other abiotic stresses, such as acid stress, hyperosmotic stress, oxidative stress, and alkali stress, has been identified (Dragosits et al. 2013; Reyes et al. 2013; Horinouchi et al. 2017b). For example, an *n*-butanol-tolerant strain obtained by Dragosits et al. (2013) also exhibited tolerance to NaCl and low pH. This study found that mutations in iron-related genes represent a common genetic factor that drives bacterial tolerance across multiple stresses. Such cross-stress observations are important because they can provide insight into the pleiotropic effects of alcohol on cellular functions.

In addition to cross-stress behavior, the acquisition of stress tolerance in bacterial cells is sometimes accompanied by “fitness cost”, a reduction of fitness (growth or viability in this case) in the absence of stress. This phenomenon is well studied in the field of antibiotic resistance in bacteria (Lenski 1998; Andersson and Hughes 2010). Fitness in the absence of stress is important for biofuel production and alcohol tolerance. One possible approach to overcoming this problem is improving fitness in the culture condition (e.g., medium, temperature, scale of culture), which is not dependent on the presence or absence of alcohol stress. Several studies demonstrated the improvement the fitness in the absence of alcohol stress via ALE (Conrad et al. 2010; Jiang et al. 2012; Zhao et al. 2013).

## Engineering alcohol tolerance

Based on information about target genes and metabolites obtained via omics analyses, it is possible to rationally engineer alcohol-tolerant bacterial strains. As described previously, several reports noted a relationship between alcohol tolerance and specific amino acids (Gonzalez et al. 2003; Horinouchi et al. 2010, 2017a; Wang et al. 2013; Haft et al. 2014). Based on these results, several groups investigated the biosynthesis or supplementation of these amino acids for improving alcohol tolerance (Gonzalez et al. 2003; Horinouchi et al. 2010, 2017a; Haft et al. 2014). In addition, it is possible to identify mutations that contribute to alcohol tolerance via genome re-sequencing analyses of tolerant strains obtained using ALE, and it is possible to evaluate the effects of specific mutations on alcohol tolerance using genome-editing technology (Pósfai et al. 1999; Wang et al. 2009; Jiang et al. 2013). Indeed, there are many examples in which alcohol tolerance has been improved by introducing mutations identified by ALE and genome re-sequencing into the genome (Atsumi et al. 2010; Minty et al. 2011; Reyes et al. 2012; Dragosits et al. 2013; Horinouchi et al. 2015, 2017a, b).

Another approach for improving alcohol tolerance is the heterologous expression of beneficial genes in a host strain. For example, overexpressing the GroESL chaperone from *C. acetobutylicum* (Abdelaal et al. 2015) in *E. coli* enhances alcohol tolerance, most likely by stabilizing or refolding proteins that are crucial for cell metabolism and survival. Overexpressing the phasin polyhydroxyalkanoate granule-associated protein (PhaP) from *Azotobacter* sp. strain FA8 (Mezzina et al. 2017) in *E. coli* also enhances alcohol tolerance PhaP from *Azotobacter* has chaperone activity and exerts a stress-alleviating effect in recombinant *E. coli* cells (de Almeida et al. 2011; Mezzina et al. 2015). In these approaches, beneficial genes associated with alcohol tolerance (e.g., GroESL or PhaP) were found in known alcohol-tolerant species such as *C. acetobutylicum* or *Azotobacter* spp. The advancement of genome sequencing technology will provide useful information about the genetic resources of various microbial species.

A tool called global transcription machinery engineering (gTME) was developed for improving cellular phenotypes (Alper and Stephanopoulos 2007). In this approach, random mutagenesis libraries of global transcription factors are generated by error-prone PCR to reprogram transcription and obtain various phenotypes. The mixture of libraries is cultured in the presence of a stressor to enrich for stress-tolerant mutants. Via this approach, alcohol tolerance was increased in *E. coli* by modifying the RNA polymerase sigma factor σ70 (Alper and Stephanopoulos 2007) and cAMP receptor protein (CRP) (Chong et al. 2013). Chong et al. (2014) performed transcriptomic analyses of CRP-engineered mutants and single-gene knockout experiments to characterize the functions of genes related to alcohol tolerance. They demonstrated that GadX (regulator of acid resistance system), HdeB (periplasmic acid stress chaperone), and several other genes were associated with isobutanol resistance (Chong et al. 2014). They also observed that the intracellular reactive oxygen species level was lower in the engineered mutants than in control strain when facing stress. These results indicated that the engineered mutants can withstand toxic isobutanol much better than the control strain.

## Concluding remarks

The development of omics technologies has undoubtedly facilitated new insights into the mechanisms by which microorganisms tolerate alcohol. For example, recent studies using omics technologies demonstrated that biological functions and response networks related to intracellular redox states are involved in alcohol stress in several microorganisms. These biological functions could be important assets for engineering alcohol-tolerant bacteria. Although alcohol-tolerant strains have been engineered, the impact of alcohol stress at the whole-cell level is not fully understood. Such an understanding is complicated by the fact that various cellular functions are undoubtedly related to the toxicity of alcohols; likewise, controlling the biological functions related to alcohol tolerance will likely be complicated. We reason that valuable information will continue to be generated using omics technology. Although omics technologies provide new biological functions that may be related to alcohol tolerance, it is necessary to verify whether these biological functions contribute to alcohol tolerance.

Further, by combining omics approaches with ALE and gTME, we can expand our search for the phenotypes of alcohol tolerance. This will accelerate the research process by identifying novel genes, proteins, or biological functions related to alcohol tolerance. We expect that further developments in the omics space and novel methodologies will allow us to further characterize the mechanisms of alcohol tolerance.
